# Exercise to Prevent and Manage Frailty and Fragility Fractures

**DOI:** 10.1007/s11914-023-00777-8

**Published:** 2023-03-28

**Authors:** Elsa Dent, Robin M. Daly, Emiel O. Hoogendijk, David Scott

**Affiliations:** 1 Research Centre for Public Health, Equity & Human Flourishing, Torrens University Australia, Adelaide, SA Australia; 2 Institute for Physical Activity and Nutrition, School of Exercise and Nutrition Sciences, Deakin University, Geelong, Australia; 3 Department of Epidemiology & Data Science, Amsterdam UMC - Location VU University Medical Center, Amsterdam, the Netherlands; 4 Department of General Practice, Amsterdam UMC - Location VU University Medical Center, Amsterdam, the Netherlands; 5 Amsterdam Public Health Research Institute, Ageing and Later Life Research Program, Amsterdam, the Netherlands; 6 Department of Medicine, School of Clinical Sciences at Monash Health, Monash University, Clayton, Australia

**Keywords:** Exercise training, Fracture prevention, Osteoporosis, Frailty

## Abstract

**Purpose of Review:**

This review identifies exercise-based recommendations to prevent and manage frailty and fragility fractures from current clinical practice guidelines. We also critically assess recently published literature in relation to exercise interventions to mitigate frailty and fragility fractures.

**Recent Findings:**

Most guidelines presented similar recommendations that included the prescription of individually tailored, multicomponent exercise programs, discouragement of prolonged sitting and inactivity, and combining exercise with optimal nutrition. To target frailty, guidelines recommend supervised progressive resistance training (PRT). For osteoporosis and fragility fractures, exercise should include weight-bearing impact activities and PRT to target bone mineral density (BMD) at the hip and spine, and also incorporate balance and mobility training, posture exercises, and functional exercise relevant to activities of daily living to reduce falls risk. Walking as a singular intervention has limited benefits for frailty and fragility fracture prevention and management.

**Summary:**

Current evidence-based clinical practice guidelines for frailty, osteoporosis, and fracture prevention recommend a multifaceted and targeted approach to optimise muscle mass, strength, power, and functional mobility as well as BMD.

## Introduction

Due to population ageing, there are an increasing number of older adults living with chronic musculoskeletal conditions, particularly osteoporosis [1]. Fractures linked to osteoporosis — known as fragility fractures — are showing a rapid increase in incidence globally [2, 3]. Fragility fractures most commonly occur from low-trauma injury, such as a fall from standing height or lower [4–6]. Compared with younger adults, older adults with a fragility fracture have a high risk of poor outcomes, including prolonged length of hospital stay (LOS), premature mortality, and high use of healthcare resources [3, 7–9]. The elevated risk of adverse outcomes is often a consequence of co-morbidities and the geriatric condition of frailty — the latter defined as age-related physiological decline across several physiological systems [5, 8, 10–13].

Frailty is associated with a decline of musculoskeletal, sensory, and neurological systems, which in turn increases falls risk [12, 13], likelihood of hip fracture [12, 13], and low trauma fragility fractures [5]. In the clinical setting, frailty can predict response to treatment and the likelihood of adverse clinical outcomes, and thus can be used to quantify any likely harms or benefits from proposed medical or surgical interventions [14, 15]. For instance, individuals with fragility fractures can be assessed for frailty in acute care and this information may be used to guide patient care planning.

Fragility fractures and frailty are both preventable. Many of the age-related changes in musculoskeletal health and function seen with advancing age, such as loss of bone mineral density (BMD), muscle mass, strength, and function, are largely due to a lack of physical activity and sedentary lifestyles [1, 6, 16, 17]. Indeed, physical inactivity in midlife has been strongly associated with an increased risk of frailty [18] and osteoporosis [6, 19] in older age. Appropriately targeted exercise can maintain or improve BMD, muscle mass, strength, and function, which in turn are crucial to reduce functional disability and thus extend the functional lifespan of older adults [20].

This review introduces the concepts of frailty and fragility fractures, after which we provide an overview of best-practice, evidence-based exercise recommendations to prevent and manage frailty and fragility fractures from new and recently updated clinical practice guidelines (referred to herein as ‘guidelines’). Guidelines are typically developed by key organisations and provide healthcare professionals with guidance on appropriate, high-value care for older adults and people with or at increased risk of chronic conditions [21]. Our review will provide a background of the most recently available literature regarding exercise interventions for the prevention and management of frailty and fragility fractures, including falls prevention. For the purposes of this review, physical activity is distinguished from exercise — physical activity being ‘any bodily movement produced by skeletal muscles that results in energy expenditure’, and ‘exercise’ defined as ‘planned, structure, repetitive, and purposeful’ physical activity aimed to ‘maintain or improve one or more components of physical fitness’ [22]. Although this review is focused on older adults aged 65 years and over, the findings are applicable to populations who are at increased risk of frailty and fragility fractures.

## Frailty

### What Is Frailty?

Frailty is an age-related clinical condition characterised by impaired physiological functioning, an increased susceptibility to stressors, and an elevated risk of adverse clinical outcomes such as falls, premature mortality, functional decline, hospitalisation, and admission to residential aged care [14, 23, 24]. Whether frailty is the result of an accelerated ageing process or due to normal ageing combined with co-morbidities has not yet been determined. Common clinical presentations of frailty include falls resulting in hospitalisation, delirium, and sudden onset of immobility [23, 25].

Frailty is common in older adults, with prevalence rates of around 5–10% in those living in the community [14, 26]. A recent meta-analysis has reported that higher prevalence of frailty is found in hospital settings (26.8% pooled prevalence) and in long-term care facilities (51.5%) [27]. Prevalence rates are substantially higher in low-middle income countries and in immigrant populations [28–30]. The prevalence of frailty increases with age and is more common in females, yet the association between sex and the rate of frailty progression shows considerable heterogeneity between populations [31].

Frailty is generally considered a pre-disability state [32]. Major contributors to frailty include sedentary behaviour, immobility, and physical inactivity [23, 32, 33]. Other risk factors include multi-morbidity [34], polypharmacy [35], poor diet [36], and age-related weight loss (termed the ‘anorexia of ageing’) [37]. Psychosocial factors such as social isolation, low education, and depressive symptoms have also been linked to frailty development or progression [29, 30, 38], as has cognitive decline [39]. Hospitalisation also contributes to the development and progression of frailty in older adults, with recent research highlighting that both the severity of disease and the care management process (including both the care provided and the ward environment) can led to an accelerated manifestation of frailty [40].

Two main models of frailty have been proposed [14]. First is phenotypic frailty (physical frailty) which is diagnosed based upon physical characteristics. Fried’s frailty phenotype classifies frailty as the existence of three or more out of five physical components: weakness (low handgrip strength), slowness (slow gait speed), weight loss, self-reported exhaustion, and low physical activity [41]. The second model is the frailty index (FI) of cumulative deficits developed by Rockwood and Mitnitski [42, 43], which is similar in nature to a co-morbidity index and considers the multidimensional aspects of frailty — co-morbidities, psychosocial components, activities of daily living (ADLs) and instrumental ADLs (IADLs), cognition, and other measures of physical functioning. Fried’s frailty phenotype and the FI remain the two most common instruments to identify frailty today and are outlined in further detail in Box 1. Another common instrument to identify frailty is the judgement-based Clinical Frailty Scale (CFS), which is a 9-point pictorial scale (with supporting text) with scores ranging from 1 (very fit) to 9 (terminally ill) [44].

Differential diagnoses of frailty include sarcopenia and malnutrition. Sarcopenia refers to an accelerated loss of muscle mass, strength, and physical performance [33, 45]. Compelling evidence exists linking sarcopenia with low BMD and osteoporosis [46], and fragility fractures [47–49] — particularly when frailty is also present [24, 33]. Malnutrition (undernutrition) in adults is defined as insufficient nutrition to meet the body’s requirements due to underconsumption or to impaired absorption of nutrients [50]. Malnutrition is closely related to frailty, with weight loss a common criterion for both conditions [51, 52]. Frailty also overlaps with multi-morbidity and disability although it is clinically distinct from these entities [53]. Difficulties with mobility are an early indicator of developing frailty, with weight loss typically the last symptom of physical frailty to manifest [23].

Frailty is interconnected with osteoporosis. An example comes from the Hertfordshire Cohort Study (*n* = 405) which reported that individuals with osteoporosis were at increased risk of being frail [*OR* (95% CI): 2.57 (0.61 to 10.78)]; co-occurring sarcopenia with osteoporosis resulted in an even greater likelihood of frailty [*OR* (95% CI): 26.1 (3.3–218.8)] [11]. Frailty is also a known risk factor for fragility fractures. A recent study of hospitalised patients (*n* = 866) identified that for every stepwise increase in the CFS score, the likelihood of mortality increased [OR values of 1.55 and 1.88 for 30-day and 1-year mortality respectively] [15]. Collectively, these findings highlight that approaches to prevent and manage frailty should also incorporate strategies targeting osteoporosis, sarcopenia, and fragility fractures.

**Box 1:** The two major frailty assessment instruments

**Table:** 

**Fried’s frailty phenotype **[41] The frailty phenotype proposed by Fried and colleagues identifies *physical* frailty, which it recognises as a biological syndrome. An individual is classified as frail by Fried’s phenotype when three or more of the following physical components are present: 1. Weakness: low grip strength 2. Slowness: low gait speed 3. Shrinking: unintentional weight loss of 10 lb (4.5 kg) in the previous year 4. Self-reported exhaustion 5. Low physical activity: physical activity per week < 383 kcal (males), < 270 kcal (females) based on the Minnesota Leisure Time Activity Questionnaire; other alternative physical activity questionnaires are often used When 1–2 components are present, an individual is classified as ‘pre-frail’. When an individual has no components present, they are classified as ‘robust’. Settings that Fried’s phenotype has been validated for include hospital, primary care, and long-term care facilities
**Frailty index of cumulative deficits** [42, 43] The frailty index (FI) of cumulative deficits describes *multidimensional* frailty. The general premise of a FI is the more (cumulative) health deficits an individual has, the more frail they are. Typically, a list of 30 or more multidimensional health deficits is considered, which includes disability, psychosocial factors, symptoms, co-morbidities, and any deficiencies in health. The FI is expressed as a ratio from 0 (no health deficits present) to 1 (all health deficits present Example: an individual has 10 health deficits present in a list of 30 health deficit variables. Their resultant FI score is 0.33 (10/30) Although the FI is considered a continuous variable, a cut-off point for frailty has been suggested as > 0.25 [23]. Thus, the individual in the above example is classified as frail The maximum FI compatible with survival in older adults is around 0.67 [43] Although the exact list of health deficits included in a FI does not technically matter, these variables must [14, 43]: - Reflect a range of physiological symptoms - Show an increased prevalence with age without a ceiling effect - Be associated with health rather than age per se - Have a prevalence ≥ 1% - Occur infrequently in populations aged under 65 years Settings that the FI has been validated for include hospital, primary care, and long-term care facilities

## Exercise, Physical Activity, and Frailty

Exercise guidelines for frailty consistently recommended that a multi-component exercise program be prescribed, and that this should include progressive resistance training (PRT) [24, 54, 55] (Box 2). The evidence underpinning exercise as a first-line treatment for frailty — and to prevent frailty — is drawn from both systematic reviews [56–58] and evidence from clinical trials [59–61]. However, the pool of literature is small and generally only of low-moderate quality [24, 54]. Moreover, interventions for frailty predominantly focus on preventing adverse outcomes associated with frailty/physical decline, rather than reducing frailty itself [14]. Accordingly, incorporation of exercise into current clinical guidelines for frailty is predominantly consensus-based [24, 54, 55]. To improve the supporting evidence-base, several recent large-scale exercise trials have been conducted in older adults with the aim to prevent frailty, improve physical function, and prevent falls. These include the Sarcopenia and Physical fRailty IN older people: multi-componenT Treatment strategies (SPRINTT) study [61], the Vivifrail project [60], the Lifestyle Interventions and Independence for Elders (LIFE) study [59], and the Staying Upright and Eating Well Research (SUPER) study [62].

Published in 2022, the SPRINTT study is a multicomponent exercise-based clinical trial which was conducted across 11 European countries and involved 1519 community-dwelling men and women (aged 70 years and over) with either physical frailty or sarcopenia [61]. The program involved moderate intensity exercise, including aerobic exercise, resistance-training, flexibility, and balance-training, and was aided by both nutritional counselling (to optimise energy 25–30 kcal/d and protein intake 1.0–1.2 g/kg/g) and technological support [61]. Frequency of training was twice weekly at a designated centre, with participants also training at home up to four times a week. The intervention was 12 months, with a 24-month maintenance phase [61]. Benefits of participation over 36 months included reductions in the risk of incident mobility disability (defined as an inability to independently walk 400 m in < 15 min) compared with lifestyle education only [61]. However, the reductions were modest, from 46.8% incident disability in the intervention group compared with 52.7% in the control group [61]. Overall, mean exercise adherence to the centre-based and home-based training was 67% and 74%, respectively (after excluding reasons for non-participation), and mean energy and protein intake increased modestly to ~ 25 kcal/kg/d and ~ 1.1 g/kg/d, respectively (from 23.3 kcal/kg/d to 0.98 g/kg/d). For the secondary outcomes, physical performance (assessed by the Short Physical Performance Battery [SPPB] summary score) also improved in intervention group participants with a SPPB score of 3 to 7 (but not 8 or 9) at baseline (maximum SPPB score is 12, with a lower score indicating worse physical functioning) compared with the control group [61]. Most secondary outcomes (hospital admissions, mortality, the number of falls and fractures) did not change with the intervention or varied by sex, with the exception that women in the intervention experienced significant net benefits to muscle (grip) strength (after 24 months only) and muscle mass compared to controls [61].

Vivifrail is a multicomponent, individually tailored exercise program designed for the prevention of falls and frailty in older adults [60]. It is based on the World Health Organisations concept of Intrinsic Capacity [63] and includes an ‘exercise passport’ for each participant [60]. The exercise program can be unsupervised and is prescribed according to an individual’s physical function (either serious, moderate, or slight limitation) as determined by three components: gait speed, SPPB score, and an assessment of risk of falling [28, 60, 64]. Participation in the ViviFrail exercise program for 3 months was found to safely improve functional capacity (as determined by SPPB score) in Spain (across three tertiary hospitals) with frailty/pre-frailty with mild levels of either dementia or cognitive decline [64]. Similar improvements were found in older females in Mexico with low muscle strength — with gains in muscle strength, gait speed, chair-stand, and timed-up-and-go performance [28].

The US Lifestyle Interventions and Independence for Elders (LIFE) trial combined walking (with a goal of 150 min/week), resistance-training, flexibility, and balance training period [59, 65]. Study participants were 1635 adults aged 70–98 years with physical limitations (defined as an SPPB score ≤ 9) [59]. Workout frequency throughout the study duration was twice weekly at a designated centre, plus 3–4 times weekly home-based training. Participation benefits of continued participation after 2.6 years included a reduction in major mobility disability (defined as the inability to complete a 400 m walk test under 15 min) from 35.5% in the control group (health education) to 30.1% in the intervention group [*HR* (95% CI): 0.82, 0.69–0.98)] [59]. Intervention adherence (the percentage of sessions attended by participants) was 63% (73% for the control group), with the intervention group maintaining an average of 104 min more walking/resistance training activities than the control group after a 24-month follow-up period [59].

The 2022 community-based New Zealand SUPER study was designed to prevent physical frailty progression in older adults screened as pre-frail, and involved three intervention types: physical activity (10 weeks of an hour/week supervised, community-based group program involving falls prevention), a nutrition education and cooking program (8 weeks involving a 3 h session per week), and a combined intervention [62]. After 6 months follow up, both the physical activity and nutritional interventions independently improved frailty (adherence rates were 80% and 88% respectively), although there were no additional benefits observed in the combined intervention; adherence rate of the combined intervention was also substantially lower (70%) [62]. However, intervention benefits were not retained 2 years post-study, highlighting that ongoing participation is needed [62].

Importantly, although aerobic exercise is key to cardiovascular fitness, it does little to address sarcopenia or reduce age-related losses in BMD [66, 67]. Progressive resistance training (PRT) is the most effective strategy for maintaining or improving muscle mass and function, and preventing the development and progression of frailty [18, 24]. PRT is a muscle strengthening method designed to gradually and progressively increase the resistance lifted as an individual’s strength improves. Equipment used can include dumbbells, resistance bands, and resistance training machines, but for optimal results, a program should include the training principles of progressive overload and specificity [24, 54]. The benefits of resistance-based training specific to the prevention and management of frailty include improvements in muscle mass, muscle strength, function, and mobility [24, 49, 68]. Resistance-based training is particularly important for individuals who are overweight or obesity undertaking a weight loss program to combat muscle mass loss when caloric restriction in medically recommended [69]. Resistance training improvements appear to be enhanced when combined with protein or a multi-nutrient enriched protein supplement [49], although the addition benefits to muscle mass and strength over PRT alone are typically modest and usually seen in those with low initial habitual intakes and when intakes increase to around ≥ 1.2 g/kg body weight/day [23, 56, 70–72].

**Box 2:** Summary of recommendations for physical activity and exercise to prevent and manage frailty and fragility from current Clinical Practice Guidelines and Position Statements

**Table:** 

**General recommendations** 1. Provide an individually tailored, multicomponent exercise program addressing an individual’s preferences, priorities, and abilities 2. Refer to a trained exercise professional (e.g. physical therapist or exercise physiologist) 3. Discourage inactivity and prolonged sitting 4. A comprehensive management plan should be provided — and should incorporate exercise/physical activity paired with adequate nutrition, particularly sufficient dietary protein. This management plan can be informed by a Comprehensive Geriatric Assessment to uncover underlying causes of fragility and/or frailty 5. The optimal prescription for general strength/resistance training for older adults is: ≥ 2 days/week, 8–10 exercises, 8–12 repetitions per set, 1–3 sets
**Frailty prevention and management** 6. Provide a multi-component exercise program for individuals with frailty or pre-frailty 7. Refer to a supervised exercise and/or physical activity program which includes a progressive, resistance training component
**Fragility fracture prevention and management** 8. Prescribe exercise programs which include challenging balance and mobility training, weight-bearing exercise, progressive resistance-training, and posture exercises (for back extensor muscles), with a focus on functional exercises mimicking ADLs, and safe movement and lifting strategies 9. Encourage regular moderate-to-high impact weight-bearing exercise: 50–100 moderate impact loads that include unusual (or diverse) loading patters (multidirectional activities) divided into 3–5 sets of 10–20 repetitions, as tolerated 4–7d/week 10. Combine exercise with optimal nutrition, including adequate calcium, vitamin D, and dietary protein intake

Consistent recommendation = a recommendation common to most clinical practice guidelines, without any conflicting recommendations; we focused only on strong recommendations with a solid evidence base.

**Key Guideline Sources (This List Is Not Exhaustive)**

**Osteoporosis and Fracture Prevention:** African Society of Bone Health and Metabolic Bone Diseases [73]; Asia Pacific Consortium on Osteoporosis [74]’ Clinician’s Guide (the Bone Health and Osteoporosis Foundation) [75••]; Asia Pacific Consortium on Osteoporosis [74]; Egyptian Academy of bone health and metabolic bone diseases [76]; Exercise and Sports Science Australia [66]; European guidance for the diagnosis and management of osteoporosis in postmenopausal women [77]; National Osteoporosis Foundation USA) [78]; National Osteoporosis Society (supported by the British Geriatric Society) [22]; North American Consensus from the National Osteoporosis Foundation, Osteoporosis Canada, and Academia Nacional de Medicina de Mexico [79]; Too Fit To Fracture exercise recommendations [80]; UK clinical guideline for the prevention and treatment of osteoporosis [81]; and the UK consensus statement on physical activity and exercise for osteoporosis [82].

**Frailty: **Asia Pacific clinical practice guidelines [54]; Australian and New Zealand Society for Sarcopenia and Frailty Research [55]; British Columbia Guidelines & Protocols Advisory Committee [83]; the British Geriatric Society [25]; and the International Conference for Frailty and Sarcopenia Research [24, 84].

**Exercise Prescription:** American College of Sports Medicine [85, 86]; Bone Health and Osteoporosis Foundation (BHOF) as outlined by LeBoff and colleagues [75••]; Exercise and Sports Science Australia [66]; Too Fit To Fracture exercise recommendations [80]; and the UK Chief medical Officers’ Physical Activity Guidelines [87, 88].

## Fragility Fractures

Fragility fractures related to osteoporosis are common in older adults, with almost one in two women and one in four men aged over 50 years predicted to sustain an osteoporotic fracture during their remaining lifetime [3]. Common sites for fragility fracture include the hip (neck of femur) (20% of all fragility fractures), vertebrae (16%), forearm/humerus (15%), tibia (in women), and pelvis [89]. Consequences of fragility fractures can be devastating for individuals. For instance, hip fractures result in severe pain, functional decline, and increased mortality risk [77, 89]. Hip fractures also place a high burden on healthcare systems [90]. In the UK, hip fracture is the most common reason for emergency anaesthesia and surgery in older people [81]. The UK’s National Hip Fracture Database report (2020) also highlighted that 48% of older adults were still not living in their own home 120 days after hip fracture [91]. In addition, across Europe hip fractures are the most expensive of all fragility fractures, encompassing over half (57%) of costs [89].

Risk factors for fragility fractures include low BMD, sex (female), a history of falls, and prior fracture [92, 93] as well as muscle weakness, frailty, inactivity, low visual acuity, poor balance, and joint instability [5, 6]. Bone geometry, bone quality, and microstructure also influence risk of a fragility fracture [94]. Identifying and addressing risk factors are key to the prevention and management of osteoporosis and fragility fractures. Medications are available, but their effectiveness is variable (30–70% fracture risk reduction), and adherence is regularly an issue (18–75% after 1 year [95]) due in part to concerns around potential side effects [89, 96, 97]. Hence, most international guidelines also recommend exercise as first-line therapy for preventing fragility fractures.

## Exercise and Fragility Fractures

Exercise is well known to improve or maintain BMD [81] and reduce the likelihood of fragility fractures and injurious falls [77, 98, 99]. International guidelines consistently recommended multicomponent exercise for the prevention and treatment of osteoporosis [22, 66, 74, 75••, 78–82, 100] (Box 1). It is recommended that individuals participate in regular weight-bearing exercise [75••, 77, 81], and be prescribed an exercise program according to their individual abilities and needs [75••, 77, 81, 101]. Supervised exercise is ideal, with a 2022 meta-analysis highlighting that supervised exercise programs were almost twice as effective at preventing fragility fractures in adults than unsupervised programs — both overall and for major fragility fractures [99]. Benefits of supervision include improved adherence, appropriate intensity progression, and safety [102].

There are two main intervention goals for an exercise/physical activity program for the prevention of fragility fractures. The first of these is to target bone; a 2% increase in hip and spine BMD has been associated with a 15–22% and 28% reduction in the risk of hip and vertebral fractures, respectively [103]. The second is to prevent falls, given that 90% of hip fracture are the result of a fall [104].

## Exercise for Improving Bone Health

The optimal exercise prescription for fragility fracture prevention should include weight-bearing (impact) exercise with PRT and challenging balance and mobility training. Weight bearing exercise is needed to provide adequate mechanical loading (strain) on the skeletal system from which improvements in BMD can occur [105]. This is true across all age-groups and in osteoporotic populations [106, 107]. Whilst the optimal weight-bearing load is not clear, guidelines recommend that exercise eliciting loads (peak ground reaction forces) that are twice one’s body weight (BW) are needed for those at moderate to high-risk and four times BW for those at low risk of fracture [66]. It is also not clear from the literature as to how many impact loads are needed to elicit improvements in bone strength or enhance bone mass, structure, and geometry [94], but there is evidence that 50 (5 × 10) daily multi-directional impacts (hops) eliciting loads of 2.2 to 2.7 times body weight can improve in hip BMD [108]. In terms of exercise frequency, there is evidence from clinical trials [109] and meta-analyses of randomised controlled trials (RCTs) [110] that two or more sessions per week are associated with the greatest benefits to bone. Furthermore, a meta-analysis of exercise intensity in postmenopausal women (*n* = 53 trials) found that moderate to high intensity exercise was required for BMD improvement (total hip and lumbar spine BMD), with low intensity exercise and walking showing little-to-no effect [111].

In recent years, there has been an emerging body of research investigating the effects of different types of exercise loads on changes in BMD. An example is the recent Australian ‘Lifting Intervention For Training Muscle and Osteoporosis Rehabilitation’ (LIFTMOR) trial, which focuses on 8 months of supervised, ‘bone-focused’, high-intensity progressive resistance and impact training (HiRIT) across various population groups [112, 113]. The LIFTMOR program involved five sets of five repetitions (at 80–85% of one repetition maximum) for three exercises (overhead press, deadlift, and squat) with 1 min rest interval between sets [112, 113]. One recent LIFTMOR trial involved 93 men aged ≥ 45 years with low BMD and found significantly greater BMD (mean 2.8% and 4.1% improvement in trochanteric and lumbar spine BMD, respectively) [112], greater lean (muscle) mass (1.5%) [112], and improved cortical bone thickness at the medial femoral neck [113] compared with matched controls. Adherence to this high intensity, supervised training program was high (mean 77.8%) and there were few adverse events, highlighting that such an approach is safe and effective for older adults with or at risk of osteoporosis [112, 113].

Additional research from the German-based Franconian Osteopenia and Sarcopenia Trial (FrOST) trial has highlighted the benefits of low-volume/high-intensity dynamic resistance exercise (HIT-DRT) for community-dwelling older men with osteosarcopenia (osteoporosis co-occurring with sarcopenia) [114, 115]. After 12 months of HIT-DRT exercise, the intervention group showed improvements in muscle mass (3.3%) relative to controls [114] and maintained BMD — whilst the control group lost 2.5% BMD [115]; both the intervention and control groups received nutritional supplementation with vitamin D, calcium, and whey protein [114, 115]. The authors concluded that this HIT-DRT protocol was feasible (mean attendance rate was 93% for 70 sessions), time-efficient (2 × 50 min sessions per week), and safe, with no adverse effects observed during the intervention [114]. However, the study was small (*n* = 21 received supervised exercise including bi-weekly phone calls, and *n* = 22 control) [114].

Multimodal exercise has also shown promise for reducing the risk of osteoporosis. A recent example is the pragmatic ‘Osteo-cise: Strong Bones for Life’ 18-month multicomponent exercise program which involved individually tailored exercise prescription incorporating high-velocity PRT combined with multidirectional and targeted impact exercises (free weights, pulleys, and machine weights), mobility exercise, and balance training [116••]. Participants included adults aged ≥ 60 years with either low BMD and/or increased falls risk [116••]. After 12 months of participation in the *Osteo-cise* program, there were significant net benefits relative to controls to lumbar spine and femoral neck BMD (1.0 to 1.1%, *p* < 0.05), muscle strength (10 to 13%, *p* < 0.05), and physical function (timed stair climb 5%; four-square step test 6%; sit-to-stand 16%, *p* < 0.05 to < 0.001), which persisted after the 6-month ‘research-to-practice’ transition [116••].

The evidence supporting exercise for fragility fracture prevention and management comes predominantly from clinical trials in high-income countries, and therefore, it is not known whether recommendations for exercise are appropriate for lower income settings in terms of adherence and cultural acceptability. Nonetheless, the latest guidelines from developing regions (e.g. Africa [73]) have similar recommendations to those of high-income countries. To summarise, international guidelines for the prevention and management of fragility fractures consistently recommend the prescription of multicomponent exercise programs which include challenging balance and mobility training, paired with weight bearing exercise, PRT, and posture exercises [22, 66, 74, 75••, 78–82, 100]. Functional exercises mimicking ADLs, with safe movement and lifting strategies, are also recommended.

## Exercise to Target Falls Prevention

Exercise is also linked with a reduction in the likelihood of falls and injurious falls [77]. The benefits of an exercise intervention for preventing falls in community-dwelling older adults (aged ≥ 60 years) were emphasised in a 2020 Cochrane review [117••]. Several modes of exercise (typically balance and functional exercises combined with resistance training) reduced falls by 34% with moderate-certainty evidence (11 RCTs and 1374 participants), and balance and functional exercises reduced fall rate by 24% compared with control (high certainty evidence; 29 RCTs, 7920 participants [117••]. Subgroup analysis revealed that the impact of effective exercises on falls prevention was the same regardless of age (participant age 75 years and over), whether or not a health professional delivered the intervention, falls risk was an inclusion criteria, or if the exercise was group or individual based [117••]. There was an insufficiency of evidence supporting the effect of resistance training alone, walking, and/or dancing on falls prevention [117••]. Similarly, a 2019 systematic review of community-dwelling older adults 60 years and over found that exercise consistently prevented falls (108 studies), with 90% of trials involving mostly female participants [118]. Importantly, the overwhelming majority of research studies demonstrate that exercise programs targeting fall reductions are safe [75••], and that falls prevention exercises can be successfully incorporated into daily life [119].

There is also a growing body of evidence indicating that exercise targeting falls prevention can also prevent fractures in older adults [120]. For example, a meta-analysis of 283 trials reported that compared with usual care, exercise alone or in combination with other fall-prevention interventions was found to be effective at preventing injurious falls; combined interventions with exercise included supplementation (either with calcium or vitamin D), treatment for vision impairment, environmental modification, clinical improvement strategies (case management), and Comprehensive Geriatric Assessment [120]. However, there are relatively few exercise-based studies which look at fracture endpoints, and the majority of these involve postmenopausal women only [75••]. Moreover, to date, there are no adequately powered RCTs of exercise with fracture outcomes [121]. Epidemiological research has highlighted that to show a relationship between exercise and fracture prevention, trials with fractures as the primary outcome are needed with over 7000 participants needed for this to be sufficiently statistically powered [122].

## Conclusions

This review provides a brief overview of current clinical practice guidelines and the most-recently available evidence on exercise for the prevention and management of frailty and fragility fractures, including falls prevention. These findings can be used by policy makers, healthcare professionals, and consumers to inform decision making regarding exercise for older adults with or at increased risk of frailty and fragility fractures. We need to do more of what works and explore how to best implement evidence-based program into real-world settings. There is sufficient evidence, supported by clinical practice guidelines, that we need to focus attention on implementing exercise interventions given their proven effectiveness for multiple musculoskeletal health outcomes. For optimal benefits, exercise programs need to be personalised based on each person’s medical history, health status, preferences, and priorities. Future research should focus on how to cost-effectively implement exercise interventions into daily life, including how to increase uptake and adherence to such programs. Appropriately funded long-term studies with patient-centred outcomes and fracture as a primary outcome are needed.
